# The ATP Binding Cassette (ABC) Transporter Gene Family in Lotus (*Nelumbo* Adans.): Genome-Wide Survey, Characterization and Gene Expression Profile

**DOI:** 10.3390/biology15060469

**Published:** 2026-03-14

**Authors:** Yumeng Zhao, Lijie Cui, Qingqing Liu, Jingjing Huo, Houchen Zhang, Dasheng Zhang, Hong Zhang

**Affiliations:** 1 College of Life Science, Ningxia University, Yinchuan 750021, China; zhaoyumeng331@163.com; 2 College of Life Science, Shanghai Normal University, Shanghai 200234, China; cuilj@shnu.edu.cn (L.C.); huojingjing0323@163.com (J.H.); 3 Shanghai Key Laboratory of Plant Functional Genomics and Resources, Shanghai Chenshan Botanical Garden, Shanghai 201602, China; nicecocoliu@sina.com; 4 Chenshan Scientific Research Center of CAS Center for Excellence in Molecular Plant Sciences, Shanghai 201602, China; 5 College of Biology and Environmental Sciences, Jishou University, Jishou 416000, China; z18799178254@163.com

**Keywords:** ABC transporter, carotenoids, expression profile, *Nelumbo*

## Abstract

ABC transporters (ATP-binding cassette transporters) constitute one of the largest known protein families and are widely distributed in plants. Their primary function involves utilizing energy derived from ATP hydrolysis to transport substrates across membranes against concentration gradients. These transporters play crucial roles in the translocation and accumulation of metabolites, stress tolerance, disease resistance, and plant defense. Lotus is an important traditional Chinese medicinal herb and contains active ingredients primarily composed of secondary metabolites, whose transport and accumulation require the involvement of ABC transporters. However, the function of these ABC transporters remains unexplored in lotus. In this study, 122 ABC transporter genes were predicted within the lotus genome. We identified 1~15 conserved motifs among the NnABC proteins and most of them were stable proteins predominantly located on the plasma membrane with ExPASy-ProtParam, ProComp and WoLF PSORT analysis. Phylogenetic tree analysis revealed that the lotus ABC transporter gene family could be divided into eight subfamilies, from ABCA to ABCI, and the evolution was predominantly driven by purifying selection. Comparative transcriptome analysis between the cultivar ‘Yindu Zhimi’ with orange-reddish stamen and ‘Weishan Hong’ with yellowish stamen, along with quantitative real-time PCR results, showed that the *NnABCG25* gene is highly specifically expressed in the orange-reddish stamen. Molecular docking demonstrated that NnABCG25 has a stable affinity for lycopene, β-carotene and β-apocarotenal, suggesting its potential involvement in the transport of carotenoids in the stamen. These findings expand our understanding of the role of ABC transporters in the transport and accumulation of carotenoids, as well as providing a valuable reference for research on the ABC transporter gene family in other plants.

## Simple Summary

Carotenoids are a class of important lipophilic pigments widely distributed in nature, which are C40 or C30 terpenoid compounds possessing an isoprene skeleton. Although extensive studies have been conducted on the biosynthetic pathways and regulatory mechanisms of carotenoids, their transport mechanisms remain poorly understood. In this study, we focused on the mutant cultivar ‘Yindu Zhimi’ with orange-reddish stamen to unravel ABC transporter function in carotenoid transport. In total, 122 NnABC transporters were identified in the lotus genome and NnABCG25 was revealed as a stamen-specific protein to mediate carotenoid transport. These findings expand our understanding of the role of ABC transporters in the transport of carotenoids, as well as providing a valuable reference for research on the ABC transporter gene family in other plants.

## 1. Introduction

ATP-binding cassette (ABC) transporters are widely distributed in both prokaryotes and eukaryotes, representing one of the largest and most functionally diverse families of transmembrane transporter proteins. In plants, they participate in various processes such as hormone transport, transmembrane transport of secondary metabolites, stress responses, and developmental regulation [1,2,3]. Based on structural features, they can be classified into three types: full-sized, half-sized, and soluble (incomplete or divergent) transporters [4,5]. A typical ABC transporter consists of four main domains: two transmembrane domains (TMDs) that form the transmembrane channel and two cytoplasmic nucleotide-binding domains (NBDs). The TMD consists of several transmembrane α-helices that regulate the specific recognition of substrates [6]. The NBDs contain three conserved signature motifs: Walker A, Walker B, and the ABC signature sequence (also known as Walker C). Full-sized ABC transporters contain two NBDs and two TMDs; half-sized transporters possess only one NBD and one TMD, with the functional transporter being achieved through homo-dimerization or hetero-dimerization of the half-molecules [3,7]; soluble types lack transmembrane domains and are composed exclusively of nucleotide-binding domains. Most ABC transporters can bind ATP and utilize the energy released from ATP hydrolysis to drive the transmembrane transport of substrates [8]. Based on sequence homology and structural characteristics, plant ABC transporters are classified into eight subfamilies: ABCA, ABCB, ABCC, ABCD, ABCE, ABCF, ABCG, and ABCI (subfamilies A–G and I), which are consistent with the classification system in animals. However, the ABCH subfamily has not yet been identified in plants [4,9].

The ABCA subfamily in plants has relatively fewer members, including both full-sized and half-sized types. In Arabidopsis, AtABCA9 was found to mediate lipid transport during seed development, with its overexpression promoting the biosynthesis of triacylglycerol (TAG) in seeds [10,11]. The ABCB subfamily, also known as P-glycoprotein (P-gp) or multidrug resistance protein, constitutes the second largest subfamily within the plant ABC transporter family [12]. ABCB proteins can efflux various endogenous and exogenous compounds, playing critical roles in polar auxin transport, the secretion of secondary metabolites, and detoxification through the elimination of harmful substances in plants. For instance, AtABCB1 and AtABCB19 can mediate polar auxin transport in Arabidopsis, thereby influencing developmental processes such as hypocotyl elongation and gravitropism [13]. Meanwhile, some ABCB proteins are also associated with herbicide resistance [14]. The ABCC subfamily belongs to full-length molecular transport proteins, also known as multidrug resistance-associated proteins (MRPs), most of which are localized to the tonoplast. ABCC transporters typically translocate glutathione-conjugated substrates (such as toxic compounds from plant metabolism or heavy metal chelates) from the cytoplasm into vacuoles, thereby playing roles in cellular detoxification and heavy metal tolerance [5]. Additionally, ABCC transporters also function in the transport of metabolites. For instance, ZmMRP3 in maize and VvABCC1 in grape are involved in the vacuolar transport of anthocyanins [15,16]. The ABCD subfamily, also known as peroxisomal membrane proteins, is responsible for transporting substrates such as long-chain fatty acids from the cytosol into peroxisomes for β-oxidation [17]. The ABCE, ABCF, and ABCI subfamilies are soluble members of the ABC transporter family, possessing only conserved NBD sequences without transmembrane domains. They do not directly mediate transport functions but play important roles in RNA metabolism and protein translation regulation through interactions with other proteins [18,19]. The ABCG subfamily represents the most numerous and functionally diverse group of ABC transporters in plants. Most ABCG proteins are localized to the plasma membrane, where they play critical roles in transmembrane transport of substances. These proteins mediate the transmembrane transport of various secondary metabolites and signaling molecules, with multiple members having been identified as transporters for abscisic acid (ABA), strigolactones, terpenoid hormones, and terpenoid alkaloids [20]. In epidermal tissues, ABCG transporter proteins mediate the efflux of hydrophobic molecules such as cuticular wax and lignin precursors across cell membranes, forming a chemically defensive surface layer [21,22]. Numerous ABCG proteins not only participate in hormone transport regulation but also play roles in pathogen defense. For instance, AtABCG25 and AtABCG40 specifically mediate the efflux and uptake of abscisic acid (ABA), respectively, thereby modulating stomatal closure and stress responses [23,24]. In tobacco, NtPDR1 is involved in chemical defense through the efflux of antifungal diterpenoids [25].

**Figure 1:**
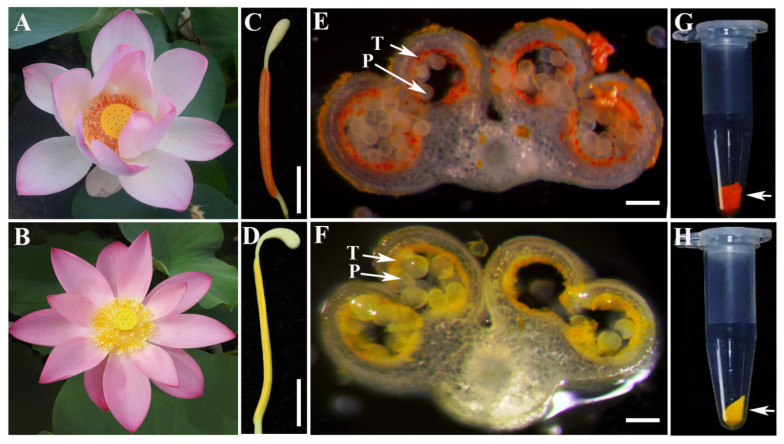
The phenotype of flower and anther of *N. nucifera* ‘Yindu Zhimi’. (**A**) The flower of *N. nucifera* ‘Yindu Zhimi’; (**B**) the flower of *N. nucifera* ‘Weishan Hong’; (**C**) individual stamen of *N. nucifera* ‘Yindu Zhimi’, consisting of an orange-reddish anther in the middle; (**D**) individual stamen of *N. nucifera* ‘Weishan Hong’, consisting of a yellowish anther in the middle; (**E**) the cross-section of the orange-reddish anther of *N. nucifera* ‘Yindu Zhimi’, with arrows indicating the tapetum and pollen, respectively; (**F**) the cross-section of the yellowish anther of *N. nucifera* ‘Weishan Hong’, with arrows indicating the tapetum and pollen, respectively; (**G**) the arrow indicates the mature orange-reddish pollen of *N. nucifera* ‘Yindu Zhimi’ in the tube; (**H**) the arrow indicates the mature yellowish pollen of *N. nucifera* ‘Weishan Hong’ in the tube. T, tapetum; P, pollen; bar = 50 mm in (**C**,**D**); bar = 100 μm in (**E**,**F**).

Although significant progress has been made in the ABC transporter gene families in various plants, research on ABC transporters in lotus (*Nelumbo* Adans.) remains unexplored. Lotus, also known as sacred lotus, is a perennial aquatic herbaceous plant belonging to the genus Nelumbonaceae. It is a globally renowned aquatic plant valued for its ornamental, edible, and medicinal properties. The lotus stamens (i.e., dried anthers), leaves, seeds, seed embryos (i.e., plumules), and receptacles were well-known traditional Chinese medicines and were recorded in the *Pharmacopoeia of the People’s Republic of China* (2020 edition, https://ydz.chp.org.cn/). Their therapeutic effects included strengthening the spleen to arrest diarrhea, cooling blood to stop bleeding, tonifying the kidneys to reduce seminal discharge, and nourishing the heart to calm the spirit. The active constituents primarily consisted of flavonoids, alkaloids and terpenoids [26,27]. Currently, there are over 2000 lotus cultivars worldwide, all of which typically exhibit pale yellow stamens. However, we have discovered a mutant cultivar named ‘Yindu Zhimi’ with distinctive orange-reddish stamens (Figure 1A,C). Studies have demonstrated that this pigmentation is attributable to elevated levels of carotenoid components within the stamens, such as lycopene, β-carotene, and β-apocarotenal [28]. Cross-sections reveal that orange-reddish carotenoid components are primarily localized in the tapetum and on the pollen surface (Figure 1E,G). The biosynthetic pathway and regulatory mechanism of carotenoids have been extensively studied in horticultural crops such as tomato, citrus, and carrot. However, to date, there are few reports regarding the transport mechanisms of carotenoids. Particularly, carotenoids have been successively identified on the pollen surface of various angiosperms [29,30,31,32,33]. However, the formation mechanism of these surface carotenoids remains unclear. It is uncertain whether carotenoids are synthesized by the tapetal cells or directly produced by the pollen grains themselves. Since the sites of carotenoid synthesis and accumulation can be observed with the naked eye through a cross-section, the orange-reddish anthers and pollen of ‘Yindu Zhimi’ provide an excellent research material for investigating carotenoid biosynthesis and transport mechanisms. Therefore, we investigated the members of the ABC transporter family in lotus to elucidate the functional relationship between ABC transporters and carotenoid transport in stamens.

## 2. Materials and Methods

### 2.1. Plant Materials

*N. nucifera* ‘Yindu Zhimi’ (red and single flower, wild type from India) and ‘Weishan Hong’ (red and single flower, wild type from China) were planted in the International Nelumbo Collection (INC) located at Shanghai Chenshan Botanical Garden, Shanghai, China. Fresh stamens of approximately 9 mm in length were collected and immediately frozen in liquid nitrogen and stored at −80 °C for use.

### 2.2. Identification of the NnABC Transporter Family

The genome of Chinese Lotus was downloaded from the NCBI database (https://www.ncbi.nlm.nih.gov/datasets/genome/GCF_000365185.1/, accessed on 11 March 2026), and the amino acid sequences of Arabidopsis ABC transporter family members were obtained from The Arabidopsis Information Resource (TAIR) database (https://www.arabidopsis.org/). Subsequently, lotus ABCs were identified from the lotus genome database using Blastp alignment with Arabidopsis ABC protein sequences as query sequences. HMM models for the conserved domains of lotus ABC transporters were constructed based on the structural domain of PF00005 (Supplementary Table S1 and File S1). Then, the HMM model was employed to screen candidate protein sequences using the HMM Search tool in TBtools-II software with an E-value threshold of 1 × 10^−10^. The final identified proteins were obtained from the intersection of HMM Search and BLASTP results. Finally, the CDD-search (https://www.ncbi.nlm.nih.gov/Structure/cdd/wrpsb.cgi, accessed on 11 March 2026) was employed to identify members of the ABC transporter protein family in lotus.

### 2.3. Physicochemical Properties and Spatial Structure Prediction of NnABC Transporters

The physicochemical properties of NnABC transporters were analyzed using the online tool ExPASy-ProtParam (http://web.expasy.org/protparam/, accessed on 11 March 2026), including parameters such as amino acid sequence length, relative molecular weight, theoretical isoelectric point, instability index, grand average of hydropathicity (GRAVY), and aliphatic index. Subcellular localization was predicted using the online tools ProComp 9.0 (www.softberry.com, accessed on 11 March 2026) and WoLF PSORT (https://wolfpsort.hgc.jp/).

### 2.4. Phylogenetic Tree, Gene Structure, and Conserved Motif Analysis

The identified NnABC transporters were used to construct a phylogenetic tree in MEGA 7.0 using the Neighbor-Joining (NJ) method, with the Bootstrap value set to 1000. The same method was used to construct a phylogenetic tree of reported ABCG transporters in *Arabidopsis thaliana* and *Oryza sativa*. The structures of the NnABC transporters were analyzed using the Visualize Gene Structure function of TBtools. Conserved motifs were analyzed using the online tool MEME Suite (http://meme-suite.org/tools/meme, accessed on 11 March 2026), with the number of motifs set to 15 and other parameters kept as default settings.

### 2.5. Calculation of Ka/Ks Ratios for the ABC Transporter Gene Family

The homologous gene pairs of the ABC transporter family in *N. nucifera* were identified using MCScanX 1.0 software. Subsequently, the Ka/Ks ratios of these genes were calculated employing the Simple Ka/Ks Calculator plugin within TBtools software. The selection pressures acting on the ABC transporter family genes in *N. nucifera* were analyzed through interpretation of their corresponding Ka/Ks ratio values.

### 2.6. Transcriptome Comparison and Gene Expression Analysis

The stamens of *N. nucifera* ‘Yindu Zhimi’ and ‘Weishan Hong’ at approximately 9 mm in length were collected as transcriptome materials for RNA sequencing. Each sample was analyzed in three biological replicates. Briefly, total RNA was extracted using the polysaccharide–polyphenol Plant Total RNA Extraction Kit (TIANGEN, Beijing, China). After quality validation of the total RNA samples, library construction and RNA sequencing (Illumina platform) were conducted by Shanghai Liebing Information Technology Co., Ltd. (Shanghai, China). Raw data were subjected to quality control to remove low-quality reads. The whole-genome sequence and gene annotation files of Chinese Lotus (https://www.ncbi.nlm.nih.gov/datasets/genome/GCF_000365185.1/, accessed on 11 March 2026) were downloaded from the NCBI database. The alignment rate between sequencing fragments and the reference genome was calculated using HISAT2 (version 2.2.1). Based on genomic alignment results and annotation files, gene expression levels were quantified using the HTSeq algorithm with FPKM normalization. Differentially expressed genes between orange-reddish stamens and yellowish stamens were identified based on the criteria of log_2_FC > 1 and FDR (false discovery rate) < 0.05.

The expression levels of *NnABC* genes were analyzed based on transcriptome data. The FPKM values within the same group were averaged, and subsequently transformed using Log_2_ (FPKM + 1) to stabilize variance. A heatmap was generated using TBtools, with different colors representing the intensity of gene expression in the clustered heatmap. Candidate proteins potentially involved in carotenoid transport were identified through phylogenetic analysis and transcriptome data.

### 2.7. RT-qPCR Analysis

Total RNA was extracted from the root, stem, leaf, petal, anther, pistil, and receptacle of *N. nucifera* ‘Yindu Zhimi’ using the Plant Total RNA Extraction Kit (TIANGEN, China). Genomic DNA removal and first-strand cDNA synthesis were performed using the PrimeScript™ RT Reagent Kit with gDNA Eraser (Takara, Osaka, Japan). Quantitative real-time PCR (RT-qPCR) was conducted using SYBR^®^ Premix Ex Taq™ II (Takara, Japan) with the following amplification program: Stage 1: 95 °C for 30 s, 1 cycle; Stage 2: 95 °C for 5 s, 60 °C for 34 s, 40 cycles. *NnACTIN* was used as the internal reference gene [34], and the primer sequences used in this text are listed in Supplementary Table S2.

### 2.8. Co-Expression Analysis of NnABCG and Carotenoid Metabolism-Related Genes

Based on transcriptome data (FPKM values), co-expression analysis was conducted between the *NnABCG25* genes and key genes in the carotenoid metabolic pathway. Expression level data of *NnABCG25* and the genes involved in the carotenoid biosynthetic pathway were extracted from the transcriptome: *DXS*, *GGPPS*, *PSY*, *ZDS*, *CRTISO*, *LYCB*, *CIS*, *NCED1*, *CCD4*, and *BCH2*. The Pearson Correlation Coefficient (PCC) was calculated to determine expression correlations between genes, with statistical significance defined as *p* < 0.05. A correlation heatmap was generated using Prism 10.0 software, presented in a lower triangular matrix format where color gradients indicate correlation strength. The yellow represents positive correlation, and the purple indicates negative correlation.

### 2.9. Molecular Docking Analysis

Referring to the method of Guo et al. [35]. Briefly, using the three-dimensional structure of Arabidopsis AtABCG25 (PDB ID: 8i38) as a template, the homology modeling method was employed to reconstruct the three-dimensional structure of NnABCG25 through the SwissModel online platform (https://swissmodel.expasy.org/). With NnABCG25 serving as the receptor and β-apocarotenal, β-carotene, and lycopene as ligands, their 3D structures were downloaded from PubChem in 3D format (https://pubchem.ncbi.nlm.nih.gov/). Molecular docking simulations were performed using AutoDock Vina (version 1.1.2) to evaluate the binding affinity between ligands and the receptor based on binding energy calculations. Finally, visual analysis was conducted using PyMOL 3.1.5 software.

### 2.10. Stamen Morphological Observation

The fresh anthers at approximately 9 mm in length were collected and cross-sectioned into approximately 0.5 mm thickness slices. Then samples were immersed in 50% glycerol solution and examined under a stereomicroscope with a DP73 digital camera to determine the localization of carotenoid pigments.

## 3. Results

### 3.1. Identification and Physicochemical Analysis of the NnABC Transporter Family

A total of 122 ABC transporters were identified in the lotus genome and named based on functional annotations and sequence characteristics. According to their structural features, these members were classified into three categories: full-sized, half-sized, and soluble transporters. Among them, there were a total of 54 full-sized transporters (44.26% of the total); 48 half-sized transporters (accounting for 39.34%) and 20 soluble transporters (accounting for 16.39%). The lengths of full-sized transporters vary significantly, with the maximum length reaching 1811 amino acids and the minimum length being 1107 amino acids. The ABCG subfamily is known to be the largest ABC transporter family in plants, and a similar pattern was also observed in lotus. In this study, 55 members were identified in the ABCG subfamily, representing 45.08% of the entire family. This finding highlighted the central role of the ABCG subfamily within the ABC protein family in lotus.

Physicochemical properties of the 122 identified NnABC transporters were analyzed with ExPASy-ProtParam tools. The amino acid lengths ranged from 224 to 1811 aa, with relative molecular mass (MW) between 25.21 and 201.91 kDa, theoretical isoelectric point (pI) ranging from 4.56 to 10.8, and aliphatic index values varying between 79.12 and 113.83. Among them, NnABCA1 encoded the longest amino acid sequence (1811 aa) and had the highest molecular weight (201.916 kDa), whereas NnABCC15-2 exhibited the smallest molecular weight of only 16.232 kDa. Stability analysis indicated that 60.6% of the members were considered stable proteins with an instability index below 40. The grand average of hydropathicity (GRAVY) analysis revealed that proteins with negative hydrophilicity average values were classified as hydrophilic proteins. In this family, 77% of the proteins were hydrophobic, while all members of subfamilies D, E, and F were hydrophilic. Within the ABCI family, only NnABCI17 was identified as a hydrophobic protein. Subcellular localization prediction revealed that the majority of lotus NnABC proteins (92 in total) were localized to the plasma membrane with ProComp 9.0 and WoLF PSORT analysis. Additionally, eight, six, and three NnABC proteins were predicted to localize to the vacuole, cytoplasm, and chloroplast membrane, respectively. Two proteins were localized to the peroxisome, chloroplast, and endoplasmic reticulum. Furthermore, six NnABC proteins exhibited multiple subcellular localization characteristics (Supplementary Table S3).

### 3.2. Phylogenetic Analysis of NnABC Transporter Family

**Figure 2:**
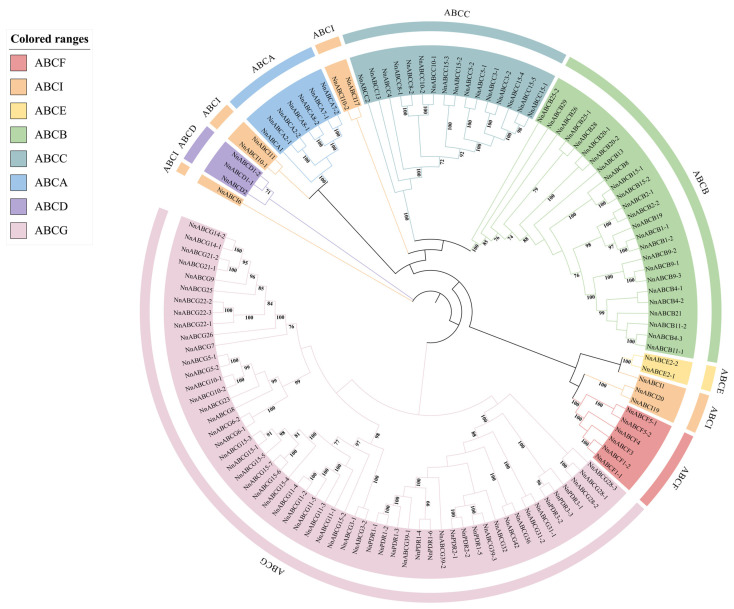
Phylogenetic analysis of 122 NnABC transporters in *Nelumbo*. The NnABC transporter family could be classified into eight subfamilies, ranging from ABCA to ABCI. The phylogenetic tree was constructed using the Neighbor-Joining (NJ) method, with a Bootstrap value of 1000.

Phylogenetic analysis revealed that the NnABC transporter family could be classified into eight subfamilies, ranging from ABCA to ABCI (Figure 2). Among these, the ABCG subfamily contains 55 proteins, representing the largest proportion at 45%, followed by the ABCB subfamily with 25 members (20.5% of the total). The ABCC subfamily comprises 16 members (13.11%), while the ABCI subfamily consists of 8 members (6.56%). The ABCA subfamily includes 7 members (5.73%), and the ABCD, ABCE, and ABCF subfamilies contain 3, 2, and 6 members, respectively. Except for the ABCI subfamily, all other subfamilies formed distinct single clades. The eight proteins of the ABCI subfamily were classified into five independent lineages, exhibiting high internal heterogeneity and significant sequence structural divergence.

### 3.3. Gene Structure and Conserved Motif Analysis of NnABC Transporter Family

**Figure 3:**
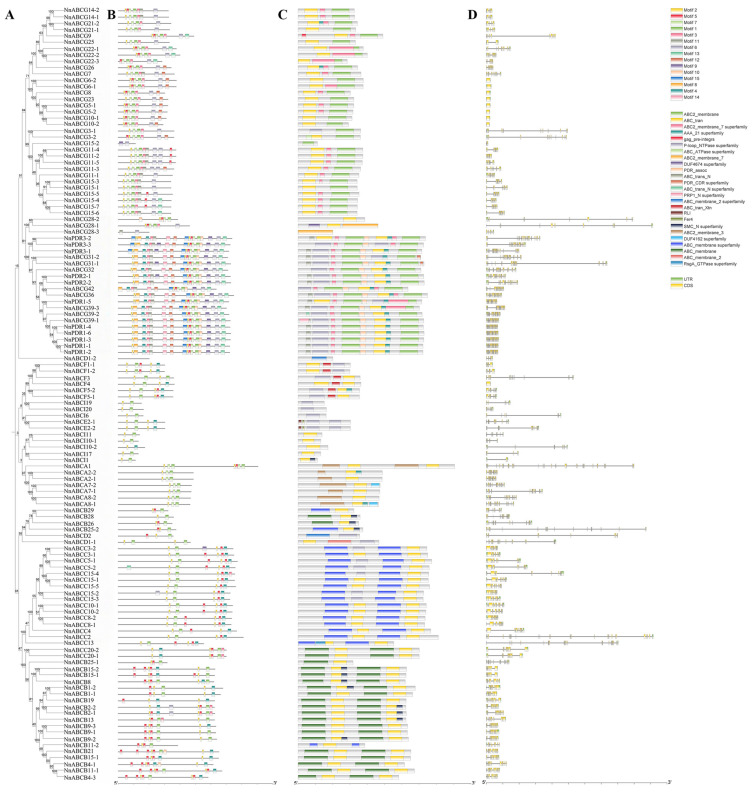
Evolutionary tree, conserved motifs, conserved domains, and gene structure distribution of *NnABC* family members in *Nelumbo.* (**A**) Phylogenetic analysis of the *NnABC* gene family; (**B**) analysis of conserved motifs in the *NnABC* gene family; (**C**) analysis of conserved structural domains in the NnABC transporter family; (**D**) gene structure and distribution of the *NnABC* family.

Given that structural analysis is crucial for understanding the functional and evolutionary relationships of homologous genes, we employed TBtools to investigate and visualize the conserved motifs, conserved domains, and exon–intron structures of the *NnABC* gene family (Figure 3). The analysis revealed that the *NnABCA1* gene possessed the highest number of exons (33 in total). In contrast, ten genes were found to contain only a single exon, including *NnABCG6-2*, *NnABCG6-1*, *NnABCG8*, *NnABCG23*, *NnABCG5-1*, *NnABCG5-2*, *NnABCG10-1*, and *NnABCG10-2* from the ABCG family, as well as *NnABCF4* and *NnABCI1*. Furthermore, intron-less sequence structures were exclusively observed within the ABCG family, encompassing *NnABCG15-2*, *NnPDR1-5*, *NnABCG9*, *NnABCG22-3*, and *NnABCG26*.

To further explore the structural diversity of the *NnABC* gene, conserved motif analysis was employed to systematically characterize its structural features. A total of 15 conserved motifs were identified within the *NnABC* gene. Integrated analysis of the conserved motifs and the phylogenetic tree showed that closely related ABC family members share highly similar motif compositions and arrangement patterns, suggesting that proteins within the same evolutionary clade may possess similar biological functions. Notably, motifs 8, 11, 12, 13, 14, and 15 were identified as unique to the ABCG subfamily, with motifs 8, 14, and 15 being exclusively present in full-size transporters of the G subfamily. In contrast, motif 1 and motif 2 were widely distributed among most members across all eight subfamilies, further highlighting the structural diversity within the NnABC transporter family. Conserved domain analysis further revealed significant differences in domain types among different subfamilies. However, members of the same subfamily typically clustered within the same branch of the phylogenetic tree and possessed identical or highly similar domain compositions, a finding consistent with the conclusions from the conserved motif analysis. To investigate the evolutionary pressure on the NnABC transporter gene family in Nelumbo, 26 pairs of homologous genes were identified using MCScanX software. The Ka/Ks analysis demonstrated that purifying selection predominantly acted during the evolutionary process of the ABC transporter gene family in Nelumbo (Table 1). In summary, the *NnABC* gene family exhibited significant diversity and subfamily-specific characteristics in terms of exon–intron structures, conserved motifs, and domain distribution. This provided crucial structural insights for subsequent functional predictions and the elucidation of evolutionary mechanisms.

### 3.4. Expression Analysis of the ABC Transporter Gene Family in Lotus Stamens

**Figure 4:**
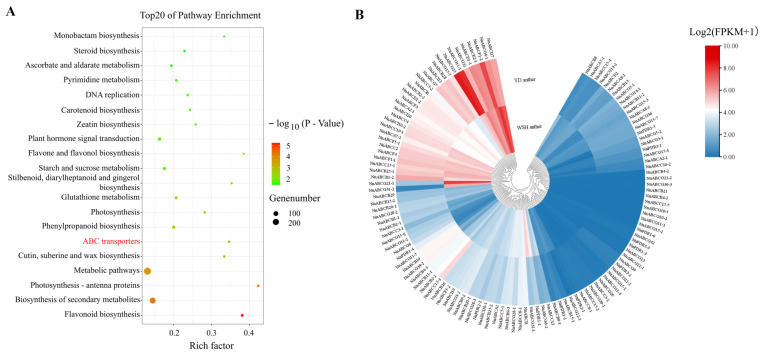
Differential expression of *NnABC* transporter genes in anther transcriptome. (**A**) Enrichment pathways of up-regulated differentially expressed genes in the transcriptome of lotus stamen; the ABC transporter group was represented in red; (**B**) heatmap of *NnABC* gene family member expression based on the transcriptome. Different colors represent different values of log_2_ (FPKM + 1).

*N. nucifera* ‘Yindu Zhimi’ was a mutant cultivar characterized by its orange-reddish stamens, whose pigment accumulation resulted from elevated levels of lycopene, β-carotene, and β-apocarotenal within the stamens. The formation and accumulation of these carotenoids primarily occured in pollen and tapetal cells [28]. To investigate the molecular mechanisms underlying orange-reddish anther development, we collected 9 mm stage anthers from two lotus cultivars ‘Yindu Zhimi’ and ‘Weishan Hong’. Comparative transcriptome analysis identified a total of 21,840 genes, with mapping rates exceeding 96% against the Nelumbo reference genome. Using thresholds of log_2_FC > 1 and FDR (false discovery rate) < 0.05, 6635 differentially expressed genes (DEGs) were identified, including 3766 up-regulated and 2868 down-regulated genes. Functional enrichment analysis revealed significant enrichment in metabolic pathways (Path: 01100), biosynthesis of secondary metabolites (Path: 01110), pentose and glucuronate interconversions (Path: 00040), starch and sucrose metabolism (Path: 00500), Zeatin biosynthesis (Path: 00908), and ABC transporters (Path: 02010) (Figure 4A). The expression heatmap analysis revealed that the majority of *NnABC* genes exhibited low expression levels in stamens. Only 37 and 32 *NnABC* genes showed relatively high expression levels (FPKM > 15) in the stamens of ‘Yindu Zhimi’ and ‘Weishan Hong’, respectively (Figure 4B). Notably, in the orange-reddish stamen, both *NnABCG25* and *NnABCG11-1* genes not only exhibited significantly up-regulated expression but also displayed the highest expression levels among all detected *NnABC* transporter genes (Figure 4B).

**Figure 5:**
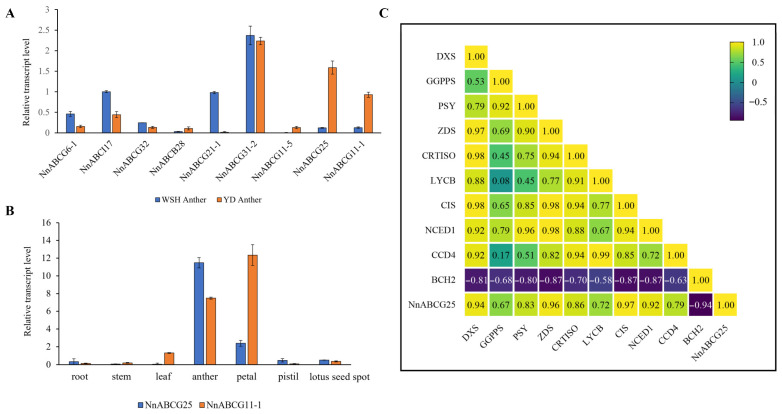
RT-qPCR analysis of ABC transporter gene in stamens and different tissues. (**A**) Differential expression of nine ABC transporter genes in lotus stamens of two different colors; (**B**) the expression profiles of *NnABCG25* and *NnABCG11-1* genes in different tissues of *N. nucifera* ‘Yindu Zhimi’; *NnACTIN* was used as internal reference gene; the error bars represent the means ± SD from triplicates (*n* = 3); (**C**) co-expression analysis of the *NnABCG25* gene and carotenoid metabolism-related genes; the yellow represents positive correlation, and the purple indicates negative correlation.

To validate the reliability of the transcriptome data, we selected nine genes with the most significant differential expression (exhibiting at least twofold or greater difference in expression levels) between two stamens for RT-qPCR analysis (Supplementary Tables S2 and S4). The results demonstrated that the expression levels were consistent with those observed in the transcriptome, confirming the reliability of the transcriptomic data. Furthermore, it was found that *NnABCG25* and *NnABCG11-1* exhibited specifically high expression levels in orange-reddish stames, with the former showing significantly higher expression than the latter (Figure 5A). To further investigate the expression profiles of these two genes, different tissues from *N. nucifera* ‘Yindu Zhimi’ were collected for RT-qPCR analysis. The expression profiles across different tissues revealed that *NnABCG25* exhibited only low expression level in roots, stems, petals, pistils, and receptacles, whereas its expression level was highest in stamens, indicating that *NnABCG25* is a stamen-specific gene (Figure 5B). In contrast, the expression pattern of *NnABCG11-1* exhibited significant differences, with its expression level in petal tissues being significantly higher than that in stamens (Figure 5B). This suggests that *NnABCG11-1* is not a stamen-specific gene, and its function might be associated with petal pigmentation or other biological processes. Based on the above findings, *NnABCG25* might play an important role in the color transition of the stamen from yellow to orange-reddish in *N. nucifera* ‘Yindu Zhimi’.

To investigate whether *NnABCG25* was involved in the carotenoid metabolism, co-expression analysis between the *NnABCG25* gene and key enzyme genes in the carotenoid metabolic pathway was conducted based on transcriptome data. The correlation heatmap revealed significant co-expression relationships between *NnABCG25* and carotenoid metabolic genes (Figure 5C). *NnABCG25* exhibited high co-expression with key carotenoid biosynthetic genes including *DXS* (r = 0.94/0.96), *PSY* (r = 0.83/0.87), *ZDS* (r = 0.96/0.97), and *CIS* (r = 0.97/0.98). In contrast, it showed a strong negative correlation with the carotenoid catabolism hydroxylase gene *BCH2* (r = −0.94/−0.82) and moderate positive correlation with the cleavage dioxygenase gene *CCD4* (r = 0.79/0.80). These results suggested that *NnABCG25* may be associated with carotenoid biosynthesis processes.

### 3.5. Prediction of the Binding Affinity Between NnABCG25 Transporter and Carotenoid Ligands

**Figure 6:**
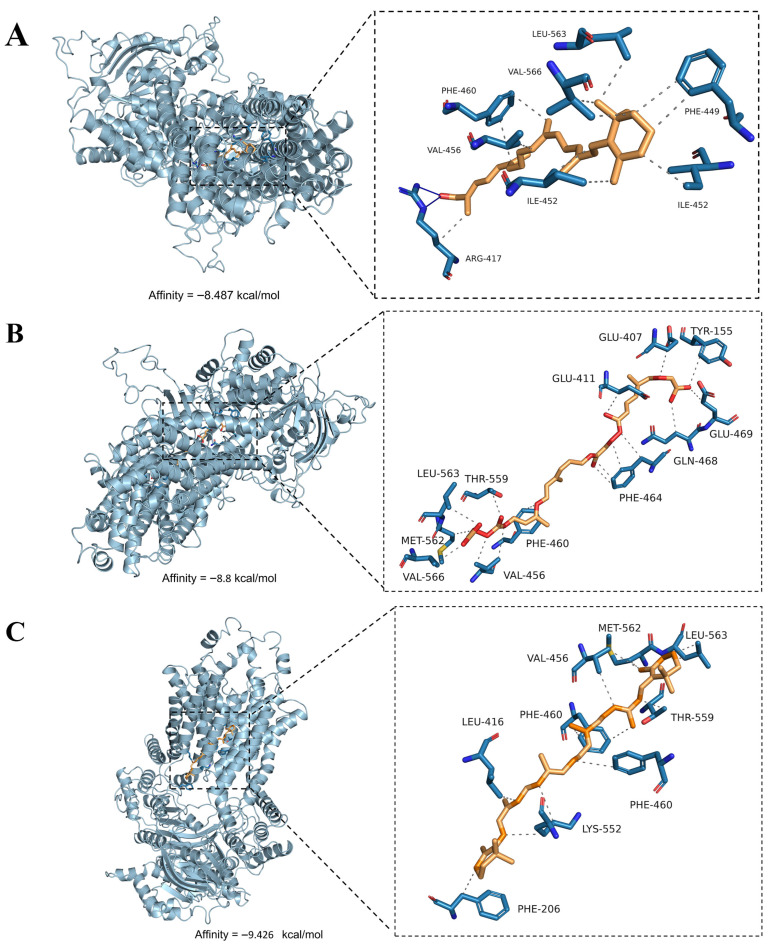
Binding affinity between NnABCG25 transporter and carotenoid ligands. (**A**) Molecular docking of β-apocarotenal to NnABCG25; (**B**) molecular docking of lycopene to NnABCG25; (**C**) molecular docking of β-carotene to NnABCG25. Hydrogen bonds are represented by blue solid lines; hydrophobic interactions are indicated by gray dashed lines. The substrate skeletons are represented in orange, and the skeletons of amino acid residues are represented in blue.

The carotenoid components in the orange-reddish stamens were primarily β-apocarotenal, lycopene, and β-carotene. To analyze whether the NnABCG25 transporter could bind to these carotenoids, we employed AutoDock Vina (version 1.1.2) for molecular docking analysis. Using PyMOL software, water molecules, unrelated ligands, and non-relevant peptide chains were removed from the original crystal structure and saved in PDB format. Subsequently, polar hydrogen atoms were added to the processed protein, and Gasteiger charges were calculated using AutoDock Tools 1.5.6. For small-molecule ligands, their conformations were first optimized through energy minimization using the MM2 force field in Chem3D 22.2 software. After exporting to PDB format, all hydrogen atoms were added, Gasteiger charges were calculated, and rotatable bonds were defined in AutoDock Tools. The docking region was defined by setting a grid box with center coordinates (center_x, center_y, center_z) of 124.809, 124.582, and 112.674, respectively, and dimensions (size_x, size_y, size_z) set at 64 Å, 68 Å, and 76 Å to ensure full coverage of the protein’s active pocket. After executing the Vina docking command, binding free energy (ΔG) values were extracted from the output log file to evaluate binding affinity. Finally, specific intermolecular interaction forces were analyzed using the online tool PLIP, and the resulting complex structures were visualized using PyMOL software. As shown in Figure 6, NnABCG25 exhibited strong binding capabilities with β-apocarotenal (−8.487 kcal/mol), lycopene (−8.8 kcal/mol), and β-carotene (−9.426 kcal/mol). Among these, β-carotene demonstrated the strongest interaction. These findings provided evidence supporting the role of NnABCG25 as a transporter protein for carotenoids.

## 4. Discussion

ABC transporters in plants are involved in the translocation of various secondary metabolites. In *Salvia miltiorrhiza*, SmABCG1 mediated the efflux of tanshinone IIA and tanshinone I into the environment [36]. In *Crocus sativus* L., CsABCC4a functioned as a crocin transporter responsible for accumulating crocin in stigma vacuoles [37]. In *Coptis japonica* Makino, CjABCB2 might participate in the transmembrane transport of berberine across cells surrounding the xylem in rhizomes [38]. Additionally, the vacuolar membrane transporter CmABCC10 identified in *Citrus medica* L. contributed to the translocation and accumulation of flavonoids [39]. Carotenoids are an important class of secondary metabolites in plants. Although their biosynthetic pathways and regulatory mechanisms have been well studied, their transport mechanisms remain unclear.

Plant pollen served as an excellent model for studying carotenoid transport. Although the accumulation of lipids and flavonoids on the surface of plant pollen has been well documented, there have also been reports indicating the presence of carotenoids on the pollen surface. However, the mechanism by which carotenoids accumulate on the pollen surface remained unclear. Since the tapetum played a crucial role in pollen development, some studies have hypothesized that carotenoids might be synthesized by tapetal cells and subsequently transported to the pollen surface through secretory pathways. These compounds were speculated to integrate with hydrophobic sporopollenin and adhered to the pollen surface, forming the complex structure of the pollen coat. However, the transport mode of carotenoids in plants has remained unclear. In the orange-reddish stamens of *N. nucifera* ‘Yindu Zhimi’, it was observed that both the pollen and tapetal cells possess the ability to produce carotenoids, thus providing an excellent cellular model for studying carotenoid formation and transport. In the orange-reddish anthers, we observed that carotenoids in the tapetal cells could be continuously released into the anther Locule along with the degradation of the tapetum. However, it remained unclear whether this process involved active or passive transport.

Given that the ABCG subfamily was proficient in transporting lipophilic molecules and participated in transmembrane transport of hormones and lipids [40], combined with the characteristics of NnABCG25, including its highly specific expression in anthers and positive correlation with key genes involved in carotenoid biosynthesis, it is hypothesized that the expression of NnABCG25 should be coordinated with the carotenoid biosynthetic process to facilitate timely transport of newly synthesized carotenoids. Molecular docking analysis further validated the strong binding affinity of NnABCG25 with key carotenoid intermediates including β-apocarotenal, lycopene, and β-carotene. We hypothesized that NnABCG25 may mediate the transport of carotenoids or their metabolic intermediates from biosynthetic sites to storage deposition zones on pollen surfaces during the critical developmental stage when anthers transition from yellow to orange-reddish coloration in ‘Yindu Zhimi’. This hypothesis aligned with established functions of ABCG transporters in mediating lipophilic pigment translocation [40]. However, the substrate specificity of NnABCG25 exhibited distinct differences compared to previously reported ABCG family members, which predominantly transport hormones or lipid compounds.

In model plants, several members of the ABCG family have been reported to play crucial roles during stamen development, which provided a valuable reference for speculating on the functions of NnABCG25 and NnABCG11-1 in this study. For example, AtABCG26 mediates transmembrane transport of lipid precursors required for pollen wall formation, and its mutation leads to pollen abortion in Arabidopsis [41]. AtABCG9 and AtABCG31 are also involved in the transport of pollen wall components, supporting the maturation of the pollen coat and thereby enhancing pollen fitness and acting synergistically with AtABCG26 [42]. Similarly, AtABCG1 and AtABCG16 have been implicated in pollen wall formation [43]. AtABCG25 functioned as a specific efflux transporter for ABA, participating in long-distance hormone transport and stress responses [44]. AtABCG11 played a central role in the synthesis of epidermal wax and cuticle [45], and it could form homo- or heterodimers with AtABCG12 to transport different cuticular components [46]. In rice, OsABCG15 might play a key role in the synthesis of sporopollenin or its transfer from tapetal cells to the anther chamber, and its loss of function led to abnormal development and male sterility [47]. In Artemisia annua, AaPDR1 was involved in the transport of the terpenoid artemisinin [48]. In maize, ZmABCG26 was homologous to AtABCG26 and participates in pollen exine formation, and its mutation led to male sterility [49]. As shown in Supplementary Figure S1, phylogenetic analysis revealed that NnABCG25 clustered with AtABCG25, while NnABCG11-1 exhibited the closest phylogenetic relationship with AtABCG11. Although NnABCG25 and Arabidopsis AtABCG25 form a monophyletic clade, they demonstrated significant functional divergence: AtABCG25 specifically mediated ABA efflux transport involved in hormone translocation and stress response without participating in carotenoid transport, whereas NnABCG25 was predicted to primarily transport carotenoids and their metabolic intermediates, thereby contributing to anther color formation. The functional diversification of these homologous proteins might represent species-specific functional evolution that occurred during the long-term evolutionary process of *Nelumbo* to meet its reproductive developmental requirements (such as the formation of orange-reddish anthers, which might be associated with pollinator attraction), demonstrating the evolutionary specificity of NnABCG25. In this study, the *NnABCG25* gene with specific high expression in lotus stamens was speculated to potentially play a similar role in carotenoid transport and deposition. In contrast, *NnABCG11-1* exhibited high expression in petals and shared closer functional characteristics with AtABCG11, presumably participating in petal cuticular wax synthesis or the transport of lipophilic metabolites associated with floral pigmentation. However, further biochemical evidence was required to substantiate these proposed transport functions. This tissue-specific expression pattern and functional differentiation not only reflected the diverse roles undertaken by ABCG family members in different organs but also provided a theoretical basis for understanding the molecular differences in pigment and structural component formation between lotus anthers and petals.

Overall, due to the limited availability of mutant variants within the ABC transporter family, current understanding of the functional roles of ABC transporters in regulating anther development remains relatively superficial. Particularly, biochemical evidence supporting the specific substrates transported by these ABC proteins is still lacking, which constitutes a limitation of this study. Compared to model plants such as *Arabidopsis thaliana* and *Oryza sativa*, research on lotus ABC transporters is still in its infancy, with no reports documenting their involvement in anther pigment transport. Through integrated analyses of phylogenetic relationships, molecular docking, and expression pattern characterization, this study preliminarily hypothesized that NnABCG25 participate in carotenoid transport. Although a direct biochemical assay was currently unavailable, this work establishes a foundational framework for subsequent functional verification experiments and addresses critical knowledge gaps in lotus ABCG transporter research.

## 5. Conclusions

This study identified 122 ABC transporter proteins in *Nelumbo* through bioinformatic analysis of the whole-genome ABC gene family, classifying this gene family into eight subfamilies. Comprehensive analyses were performed, integrating multiple aspects including physicochemical properties, phylogenetic relationships, gene structures, conserved motifs, and tissue-specific expression patterns. The number and subfamily distribution of NnABC transporter genes showed certain similarities with other angiosperms but also exhibited unique characteristics. Ka/Ks evolutionary analysis indicated that the NnABC transporter gene family predominantly underwent purifying selection. Significant diversity was observed within the NnABC transporter family in terms of amino acid length, relative molecular weight, theoretical isoelectric point, average hydrophilicity, aliphatic index, and instability index. Transcriptome screening of lotus stamens identified NnABCG25 as an anther-specific highly expressed transporter. Co-expression and molecular docking analysis revealed its association with carotenoid biosynthesis pathways, predicting its potential binding capacity with β-apocarotenonal, lycopene, and β-carotene. These findings suggested that NnABCG25 was involved in carotenoid transport and accumulation within stamens. Next, the binding activity of NnABCG25 with carotenoids will be verified by an in vitro transport assay. Heterologous expression or CRISPR of *NnABCG25* gene using the genetic transformation system in *N. nucifera* or other model plants will be performed to confirm the transport relationship with carotenoids. These findings will provide a basis for further investigation of the biological functions of ABCG subfamily members in the color transition process of lotus stamens in ‘Yindu Zhimi’ and expand our understanding of transport functionalities within plant ABCG subfamily members.

## Figures and Tables

**Table 1:** Ka/Ks of ABC transporter gene family in *Nelumbo*.

Homologous Gene Pairs	Ka/Ks	Homologous Gene Pairs	Ka/Ks
NnABCB1-1/NnABCB1-2	0.07	NnABCF5-1/NnABCF5-2	0.09
NnABCF1-1/NnABCF1-2	0.04	NnABCC8-1/NnABCC8-2	0.34
NnPDR2-1/NnPDR2-2	0.12	NnABCG21-1/NnABCG21-2	0.15
NnABCG39-1/NnABCG39-2	0.19	NnABCG31-1/NnABCG31-2	0.24
NnABCG42/NnABCG36	0.15	NnABCG28-1/NnABCG28-3	0.22
NnABCC15-2/NnABCC15-3	0.21	NnPDR3-1/NnPDR3-3	0.18
NnABCC10-1/NnABCC10-2	0.24	NnABCB9-2/NnABCB9-3	0.13
NnABCB20-1/NnABCB20-2	0.08	NnABCG11-2/NnABCG11-4	0.1
NnABCG6-1/NnABCG6-2	0.19	NnABCB2-1/NnABCB2-2	0.1
NnABCB15-1/NnABCB15-2	0.12	NnABCG14-1/NnABCG14-2	0.13
NnABCA2-1/NnABCA2-2	0.16	NnABCG10-1/NnABCG10-2	0.15
NnABCG5-1/NnABCG5-2	0.09	NnABCG28-1/NnABCG28-2	0.28
NnABCA8-1/NnABCA8-2	0.24	NnABCC15-4/NnABCC15-5	0.25

Ka/Ks > 1 indicates positive selection, Ka/Ks < 1 represents purifying selection, and Ka/Ks = 1 signifies neutral evolution.

## Data Availability

The original contributions presented in this study are included in the article/Supplementary Materials. Further inquiries can be directed to the corresponding authors.

## Supplementary Materials

The following supporting information can be downloaded at https://www.mdpi.com/article/10.3390/biology15060469/s1, File S1. HMM folder. Table S1. Protein domains of ABC transporter family. Table S2. RT-qPCR primers of candidate *NnABC* genes. Table S3. Structural and physicochemical properties and subcellular localization analysis of the ABC transporters in *Nelumbo*. Table S4. DEGs FPKM level. Figure S1. Phylogenetic tree of NnABCG transporter protein in *Nelumbo* sp. and ABCG in other species.
