# Supplementary material for: The ATP Binding Cassette (ABC) Transporter Gene Family in Lotus (Nelumbo Adans.): Genome-Wide Survey, Characterization and Gene Expression Profile
Source: Biology (Basel). 2026 Mar 14;15(6):469. doi: 10.3390/biology15060469 (PMC13023602; doi:10.3390/biology15060469)
Supplement: Supplementary file 1 [file biology-15-00469-s001.zip › Supplementary Table S1 and S2.pdf]

Table S1 Protein domains of ABC transporter family

Accession Number	Structural Domain	Accession Number	Structural Domain
PF00005	ABC_tran	PF12679	ABC2_membrane_2
PF12848	ABC_tran_Xtn	PF12698	ABC2_membrane_3
PF00664	ABC_membrane	PF12730	ABC2_membrane_4
PF06472	ABC_membrane_2	PF13346	ABC2_membrane_5
PF13748	ABC_membrane_3	PF06182	ABC2_membrane_6
PF01061	ABC2_membrane	PF19055	ABC2_membrane_7

Table S2 RT-qPCR primers of candidate *NnABC* genes

Gene Name	Primer-F (5'-3')	Primer-R (5'-3')
NnACTIN	TGATCGGAATGGAAGC	CAGCAATACCAGGGAAC
NnABCG25	CTCGATCTTGCCAACGGAGT	CTTCATGGGCCCTGTAGC
NnABCG11-1	ATGCTCAATTCACCAGCCCA	ATCAGTCCTTGCAAACC<br>GCA
NnABCG6-1	CTCGACTTGATCCGGCAACT	CGACATAGGGCTCGCATC<br>AT
NnABCI17	TGCCAATGACCCAAAGGTGT	GGACCTCAACGATCTCA
NnABCG32	CTGGTATGTGGGTGGTGGTT	ACTTGGAATGCTGTCTCT
NnABCB28	GGTGGACAGAGGCAGAGAAT	TGGGTGCCTAGTTCTGCA
NnABCG21-1	CTTGATTCCACCACGGCACA	AT
NnABCG31-2	TGGGCTGCAATCGAGAAGTT	ATTCAGGAGCCACACCA
NnABCG11-5	TTATGTTAGGCGCGAACCAGT	TTAGC
