# Supplementary figures and images for: The ATP Binding Cassette (ABC) Transporter Gene Family in Lotus (Nelumbo Adans.): Genome-Wide Survey, Characterization and Gene Expression Profile
Source: Biology (Basel). 2026 Mar 14;15(6):469. doi: 10.3390/biology15060469 (PMC13023602; doi:10.3390/biology15060469)

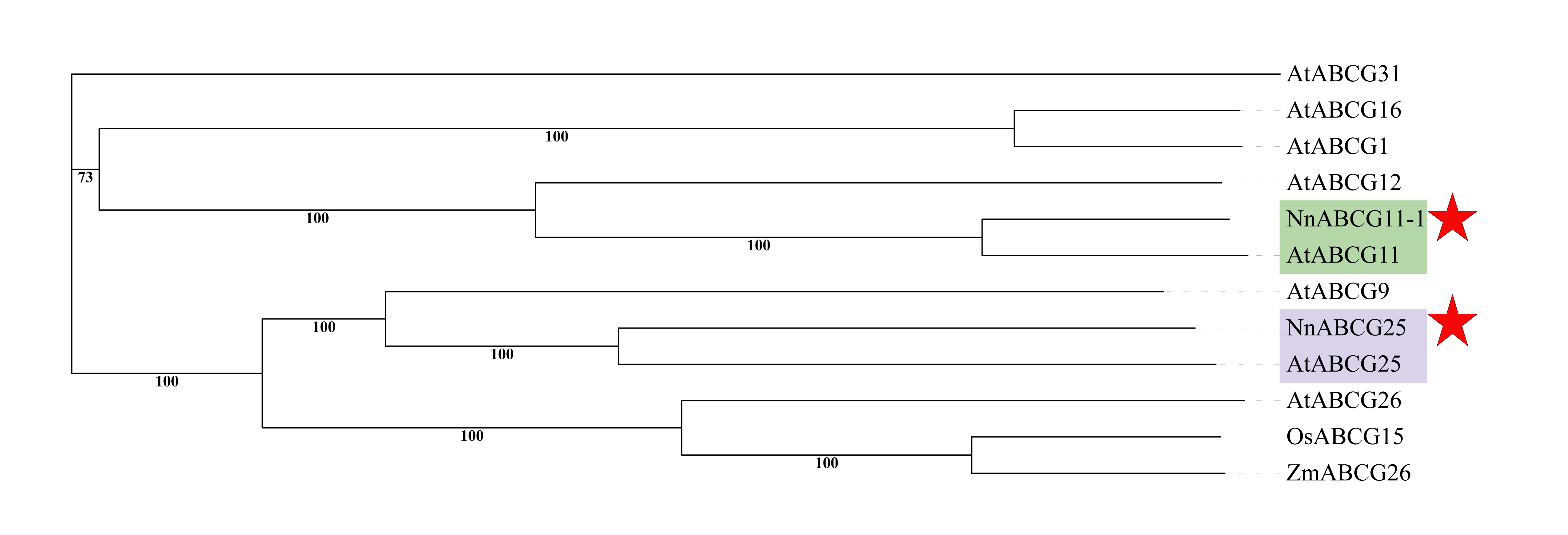

Supplement: Supplementary file 1 [file biology-15-00469-s001.zip › Supplementary Figure S1.tiff]
